# Aerogels from Chitosan Solutions in Ionic Liquids

**DOI:** 10.3390/polym9120722

**Published:** 2017-12-16

**Authors:** Gonzalo Santos-López, Waldo Argüelles-Monal, Elizabeth Carvajal-Millan, Yolanda L. López-Franco, Maricarmen T. Recillas-Mota, Jaime Lizardi-Mendoza

**Affiliations:** 1 Grupo de Investigación en Biopolímeros—CTAOA. Centro de Investigación en Alimentación y Desarrollo, A.C., Hermosillo, Sonora 83304, Mexico; gonzalosantos@estudiantes.ciad.mx (G.S.-L.); ecarvajal@ciad.mx (E.C.-M.); lopezf@ciad.mx (Y.L.L.-F.); 2 Polímeros Naturales. Centro de Investigación en Alimentación y Desarrollo, A.C., Unidad Guaymas, Guaymas, Sonora 85480, Mexico; waldo@ciad.mx (W.A.-M.); mrecillas@ciad.mx (M.T.R.-M.)

**Keywords:** aerogels, chitosan, ionic liquids, ionogels

## Abstract

Chitosan aerogels conjugates the characteristics of nanostructured porous materials, i.e., extended specific surface area and nano scale porosity, with the remarkable functional properties of chitosan. Aerogels were obtained from solutions of chitosan in ionic liquids (ILs), 1-butyl-3-methylimidazolium acetate (BMIMAc), and 1-ethyl-3-methyl-imidazolium acetate (EMIMAc), in order to observe the effect of the solvent in the structural characteristics of this type of materials. The process of elaboration of aerogels comprised the formation of physical gels through anti-solvent vapor diffusion, liquid phase exchange, and supercritical CO_2_ drying. The aerogels maintained the chemical identity of chitosan according to Fourier transform infrared spectrophotometer (FT-IR) spectroscopy, indicating the presence of their characteristic functional groups. The internal structure of the obtained aerogels appears as porous aggregated networks in microscopy images. The obtained materials have specific surface areas over 350 m^2^/g and can be considered mesoporous. According to swelling experiments, the chitosan aerogels could absorb between three and six times their weight of water. However, the swelling and diffusion coefficient decreased at higher temperatures. The structural characteristics of chitosan aerogels that are obtained from ionic liquids are distinctive and could be related to solvation dynamic at the initial state.

## 1. Introduction

Chitosan (Cs) is a natural linear polysaccharide generated from the deacetylation of chitin and is composed of β-(1-4)-d-glucosamine units and β-(1-4)-*N*-acetyl-glucosamine distributed along the polymeric chain. The physicochemical characteristics and functional properties of Cs, such as its polycationic character, biocompatibility, low toxicity, and structural capacity, make it a polysaccharide of interest in different fields. The mechanical and structural properties of chitosan allow for different types of materials to be obtained from chitosan solutions, e.g., nanostructured porous materials. Aerogels are a specific type of nanostructured porous material that are characterized by mesopores (pore diameter between 2 and 50 nm) and large surface areas. Obtaining aerogels of chitosan could improve the availability of the functional groups of chitosan [1]. Together, the aerogel characteristics with the functional properties of chitosan, potentially provides materials with multiple applications, e.g., adsorption, transport, and controlled release of bioactive molecules, toxics, and pollutants removers, among others.

Ionic liquids (IL) are an alternative medium for chitosan dissolution. When compared to the traditional aqueous acid solvents used to dissolve Cs, IL have different physicochemical characteristics because they consist of only ions and water is not needed to dissolve chitosan. Most of the studies of chitosan with IL have been focused on the formation of solutions. Imidazolium based IL, as 1-butyl-3-methylimidazolium acetate (BMIMAc), and 1-ethyl-3-methyl-imidazolium acetate (EMIMAc), have been highlighted because they are able to dissolve chitosan at high concentrations (up to 10% *w/w*). Other reports have focused on forming materials from chitosan solutions in IL e.g., fibers, blends, films, membranes, hydrogels, and ionogels [2,3,4,5,6,7,8,9,10,11,12]. Aerogels from chitin with BMIMAc solutions has been reported [13], but the information about chitosan ionogels and aerogels from IL solutions is limited.

Supercritical CO_2_ drying, unlike other types of drying, keeps most of the internal structure formed at gelation because the effects of surface tension on the three-dimensional macromolecular network are minimized. Therefore, chitosan aerogels that are generated from solutions of Cs in IL (BMIMAc and EMIMAc) were produced in order to study the physicochemical characteristics of these novel materials. Concurrently, observations on the structural features of the aerogels could be related to the network formation and molecular conformation of chitosan in ionic liquids at the physical gelling process.

## 2. Materials and Methods

### 2.1. Materials

Shrimp shells (*Pandalus borealis*) Cs was acquired from Primex (batch No. TM 1961, Siglufjörður, Iceland). The degree of acetylation (DA) of Cs was 16.3%, as determined by solid-state cross-polarization magic angle spinning ^13^C nuclear magnetic resonance spectroscopy (CP/MAS ^13^C-NMR) performed in a Bruker Avance TM 400WB (Bruker Biospin, Wissembourg, France, 9.4 Tesla, 1 ms contact time, 2000 scans) [14,15]. The weight average molecular weight (*M*_w_) of Cs was 2.01 × 10^5^ g/mol, determined by static light scattering, as described previously [16]. Chitosan was purified, as described by Rinaudo et al. (1999), before use it [17]. Commercial grade IL, BMIMAc and EMIMAc (BASF, Steinheim, Germany), were used as received just prior each use they were stored in desiccator and kept at 105° C for 24 h in vacuum to minimize moisture. High grade supercritical drying liquid CO_2_ (99.99% purity) was supplied by Infra (Hermosillo, México). All of the compounds and solvents that were used were reagent grade acquired from recognized commercial chemical distributors. The water used for the experiments was deionized type I (resistivity of 18.2 MΩ·cm at 25 °C) unless stated otherwise.

### 2.2. Solutions of Chitosan in Ionic Liquids

The moisture-free IL was added into a round-bottom flask with a sufficient amount of chitosan to obtain a 2% (*w/w*) concentration. This solution was heated in an oil bath at 105 °C under magnetic stirring at least 6 hours under a nitrogen atmosphere. Upon complete Cs dissolution it was stored in a desiccator at room temperature until use. Two types of solutions were obtained: Cs in EMIMAc (CsEMIM) and Cs in BMIMAc (CsBMIM).

### 2.3. Formation of Ionogels

The physical gels were formed using approximately 0.1 g of the Cs-IL solution that was casted in cylindrical containers (0.4 cm diameter), with the bottom being covered with plastic paraffin film. Gelation was induced by vapor diffusion of an antisolvent, such as ethanol or water, in a closed chamber [18]. The gels were recovered after 48 h and were transferred to a 70% ethanol aqueous mixture. These gels that contain IL as liquid phase confined within a polymer matrix are defined as ionogels [19].

### 2.4. Formation of Aerogels

The ionogels were thoroughly rinsed with aqueous ethanol (70%) until the IL was completely removed. The presence of IL in the rinsing media was monitored with UV/VIS spectroscopy, scanning between 190 to 300 nm using the ethanol-water mixture as reference. Afterwards, the liquid phase was replaced with acetone, which has higher miscibility with supercritical CO_2_. For this, the gels were repeatedly rinsed with a gradient of acetone-water mixtures that ended with two rinses with pure acetone. The acetogels (gels containing acetone as fluid phase) were dried with supercritical CO_2_ (>32 °C and 73 atm) in a pressurized reactor. At the end of the process, the dried aerogels were stored in a desiccator at room temperature.

### 2.5. Characterization of Aerogels

#### 2.5.1. Chemical Identity

The chemical composition of the samples was determined analyzing the characteristic bands in the infrared spectrum obtained by a Fourier transform infrared spectrophotometer (FT-IR, Thermo Scientific, Nicolet iS-50, Madison, WI, USA), using the attenuated total reflection (ATR) mode. All of the measurements were performed at room temperature collecting 32 scans at 4 cm^−1^ resolution.

#### 2.5.2. Structural Analysis

The surface area, pore volume, and nitrogen adsorption and desorption isotherms of the aerogels were determined with the surface area analyzer Nova 2200e (Quantachrome Instruments, Boynton Beach, FL, USA), and analysis of the data with the software NovaWin version 11.02 (Quantachrome Instruments, Boynton Beach, FL, USA). The morphological characteristics of the aerogels were thoroughly observed by field emission scanning electron microscopy (Hitachi SU8000, Tokyo, Japan). The images were obtained using an accelerating voltage of 1.0 KeV.

#### 2.5.3. Degree of Swelling

The swelling capacity of the aerogels at equilibrium was determined from their immersion in water at different temperatures (20, 25, 30, and 40 °C). The weight gain of the samples was periodically monitored by removing the excess of water with filter paper. Equation (1) was used to estimate the degree of swelling (*W*), as follows:

(1) $$W=\frac{P\;-\;P_{0}}{P_{0}}=\frac{P}{P_{0}}\;-\;1$$

where *P*_0_ is the weight of the dry aerogel and *P* is the weight of the aerogel in the wet state [20].

## 3. Results and Discussion

The vapor diffusion of a non-solvent agent was useful to produce ionogels from Cs solutions in IL. By this way, it was possible to obtain three different types of physical chitosan ionogels (Table 1). Using ethanol as non-solvent agent gels were obtained from both Cs-IL solutions. These gels were clear, rigid, and brittle; the only noticeable difference among them was the color tone, darker yellow for the gels from CsBMIM. It has been indicated that the main effect of IL on polysaccharides is to disrupt the hydrogen bonds and promoting their dissolution. Low molecular weight alcohols are miscible with imidazolium based ionic liquids [21], but chitosan does not dissolve in alcohols. Therefore, when ethanol diffuses into a Cs-IL solution, the solvation effect of the ionic liquid over chitosan decreases, favoring the interactions between chitosan chains and subsequently leading to the generation of a gel. Conversely, water vapor was only useful to produce gels from the CsEMIM solution. It has been noticed that BMIMAc has a lower affinity for water than EMIMAc [22], this could be related to lower diffusion rates that do not decrease its chitosan solvation capacity in preventing the gel formation. It should be taken in account that chitosan do not dissolve in water, but it is hygroscopic. As result, the obtained gels from water diffusion were weak and difficult to manipulate without compromising their integrity. Hence, subsequent procedures and analysis were performed using only ionogels that are produced by ethanol treatment.

**Figure 1:**
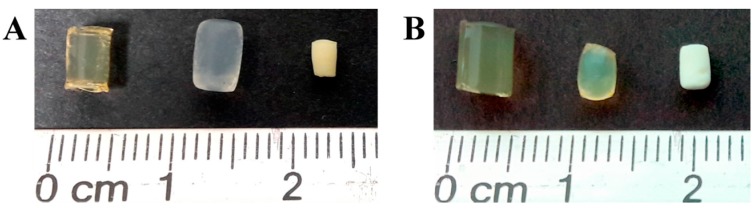
Materials formed from the Cs-IL solutions: (**A**) CsE and (**B**) CsB. From left to right: ionogel, acetogel and aerogel.

When the ionogels were rinsed with an ethanol-water mixture they became more translucent, reducing their yellow color (Figure 1). The continuous rinsing of the gels gradually eliminated the ionic liquid from inside the gels. The ethanol concentration that was used allowed for keeping the ionogels volume without causing drastic swelling or shrinkage. In the subsequent fluid phase replacement with acetone, the CsE ionogels decrease 25% their volume, and in the case of CsB, the reduction was 42%. Apparently, the chitosan chains underwent rearrangement within the formed network to a more stable configuration as a result of the interaction with acetone [23].

The obtained aerogels were rigid and brittle cylinders with opaque white color (Figure 1). The volume reduction when compared to the starting ionogels was of 73% for CsE and 82% for CsB. This behavior is similar to that reported for aerogels obtained from chitosan and κ-carrageenan [24]. Such volume reduction has been associated with the rearrangements of chitosan chains due to their lower affinity for acetone. This molecular movement does not cease until all of the acetone has been removed, even when the supercritical CO_2_ drying reduce the effects of surface tension in the material [25].

### 3.1. Characterization of Chitosan Aerogels

**Figure 2:**
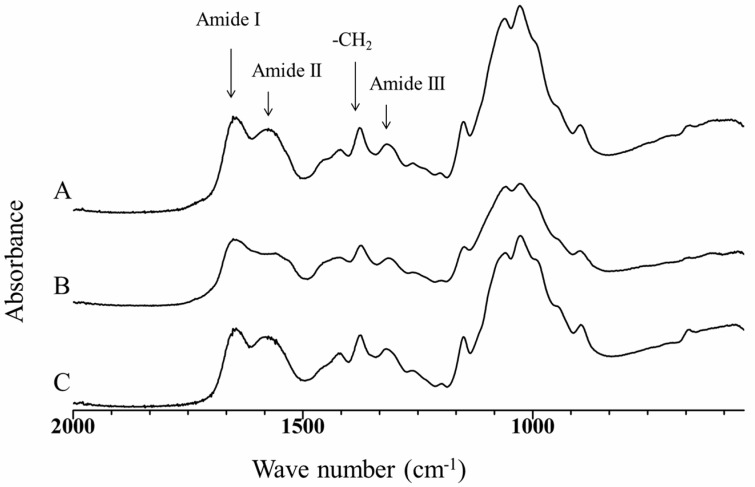
Fourier transform infrared spectrophotometer (FT-IR) spectra of (**A**) CsE aerogel; (**B**) CsB aerogel and (**C**) chitosan.

The infrared spectra (2000–500 cm^−1^) of chitosan and the obtained aerogels are shown in Figure 2. The main characteristic bands of chitosan are also observed in the aerogels spectra. At 1652 cm^−1^ is observed the stretching vibration band of the C=O bond that is associated to the amide I; the amide II –NH_2_ deformation is related to the band at 1580 cm^−1^; the band at 1424 cm^−1^ is associated to the CH_2_ bending; at 1380 cm^−1^, the symmetrical vibration deformation of the CH_3_ group is observed; the band at 1318 cm^−1^ is associated to the amide III; the antisymmetric tension mode of the COC bridge is observed at 1150 cm^−1^; finally, the fingerprint zone, between 1075 and 1026 cm^−1^, is characteristic of the polysaccharides [26,27]. There is no evident modification on the spectra that indicate chemical changes in the chitosan as result of the gel formation or the drying process. Furthermore, there are not absorbance bands that could be related to the presence of residual IL in the aerogels.

**Figure 3:**
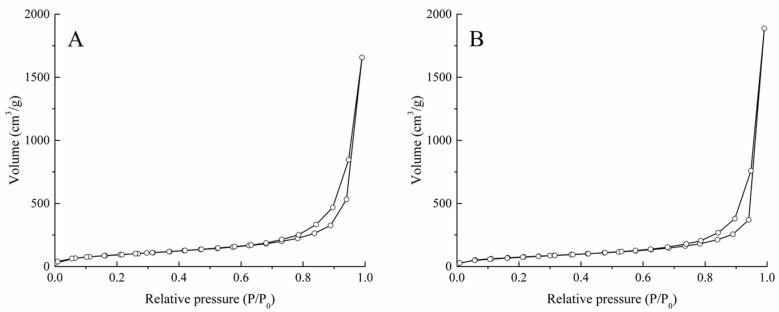
N_2_ adsorption and desorption BET isotherms of (**A**) CsE and (**B**) CsB aerogels.

The N_2_ adsorption and desorption isotherms of both types of aerogels are classified as type IV according to the IUPAC conventions (Figure 3). The observed hysteresis of N_2_ desorption at high relative pressures is indicative of a mesoporous dry material [28]. The specific surface area (*S*_BET_) and pore volume that are calculated from the adsorption and desorption isotherms are included in Table 2. The specific surface areas of the aerogels are in the higher rank when compared with other pure polysaccharide aerogels [24,29,30]. The pore size obtained was within the range of mesopores, which is characteristic of aerogels.

**Figure 4:**
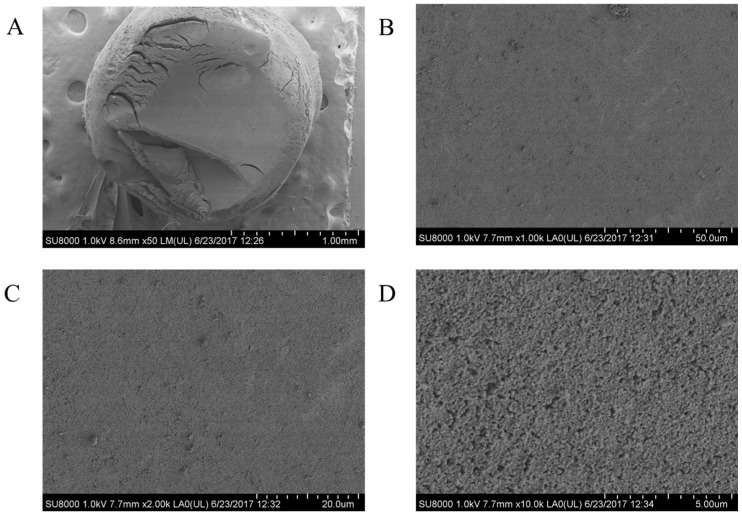
SEM images of the CsE aerogels (**A**) 50×; (**B**) 1000×; (**C**) 2000×; and, (**D**) 10,000× magnification.

**Figure 5:**
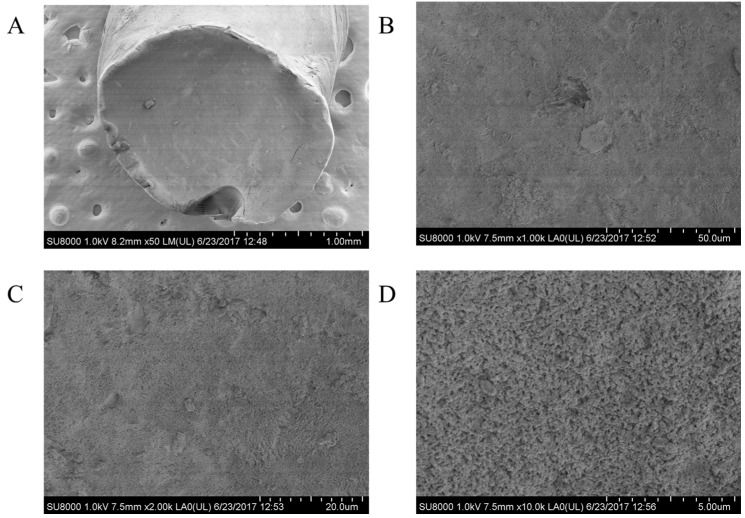
SEM images of the CsB aerogels. (**A**) 50×; (**B**) 1000×; (**C**) 2000×; and, (**D**) 10,000× magnification.

The scanning electron microscopy (SEM) images of the aerogels are shown in Figure 4 and Figure 5. Both of the aerogels appear as uniform materials with some imperfections, which could be caused by fracture events. At the highest magnification available (×10,000), the internal structure of the aerogels looks as aggregated clumps forming a compact network with heterogeneous pores. The appearance of the pores is consistent with the mesoporous characteristics of the aerogels. The main difference between both types of aerogels is that the internal structure of CsB appears to be denser. The internal structure of the aerogels produced from ionogels is different to previously reported chitosan aerogels [24,29]. These differences could be related to the solvent-polymer interaction. Electrostatic repulsions dominate in the aqueous acid chitosan solutions. In such conditions, the polysaccharide molecules adopt an extended hydrodynamic volume conformation [17]. Conversely, these repulsive forces are absent in Cs-IL solutions; thus, the chitosan molecules have relatively smaller dimensions, generating more compact structures in their aerogels.

### 3.2. Diffusion Properties of Aerogels

**Figure 6:**
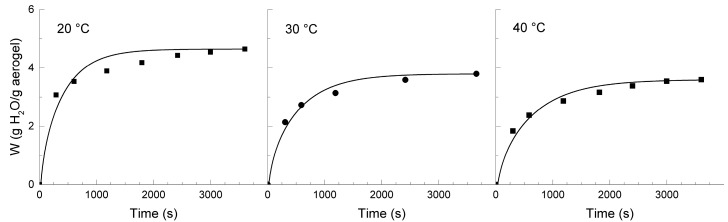
Swelling curves of the CsE aerogels in water at different temperatures (20, 30, and 40 °C). Experimental data (points) and adjustment (lines) with Equation (2) are included.

**Figure 7:**
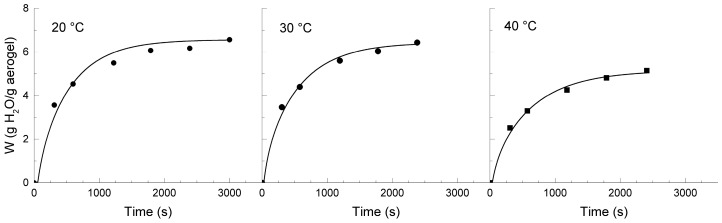
Swelling curves of the CsB aerogels in water at different temperatures (20, 30, and 40 °C). Experimental data (points) and adjustment (lines) with Equation (2) are included.

The physical characteristics of aerogels, such as their large surface area and mesoporosity, endow these materials with a large capacity to adsorb certain compounds. The diffusion properties are key to evaluating the performance of these materials for important applications in pharmacy and biotechnology, among others. For this purpose, dried aerogels that are obtained from chitosan solutions in EMIMAc and BMIMAc, as described previously, were swollen in water at different temperatures between 20 and 40 °C and their kinetics was followed. In Figure 6 and Figure 7, the experimental data is presented.

The CsE aerogels absorbed between three and four times their weight and exhibited a decreasing swelling capacity with an increasing temperature. In contrast, the CsB aerogels showed a greater capacity of absorption (between five and six times their weight), and the effect of temperature on the swelling capacity seems to be less marked. When compared with the aerogels of chitosan that was obtained from aqueous acid solutions, the aerogels from chitosan in IL showed higher *W_∞_*. Previous studies with chitosan or chitosan-polyelectrolyte complex matrices that were prepared from aqueous media have shown a similar tendency to decrease the swelling capacity with temperature [20,31].

The Fick’s law equation resolved for diffusion through a circular cylinder of radius *r*, keeping the diffusant concentration constant, becomes [32]:

(2) $$\frac{W}{W_{\infty }}=1-\overset{\infty }{\underset{n=1}{\sum }}\frac{4}{\;r^{2}\alpha_{n}^{2}}\exp\left(-D\alpha_{n}^{2}t\right),$$

in which *W* is the swelling degree at time *t*, and *W_∞_* is the corresponding quantity at equilibrium, $\alpha_{n}^{2}$ are the *n* first positive roots of the Bessel function of the first-kind, and *D* is the diffusion coefficient.

Equation (3) was solved for the first 15 terms of the summation and the diffusion coefficients that were adjusted through a non-linear least square fitting process. In all situations, satisfactory adjustments were obtained from Equation (2), as could be appreciated from the curves that are traced in Figure 6 and Figure 7. The estimated values of *D* are summarized in Table 3. The diffusion coefficients are similar for both types of aerogels and compare to those that are reported for swelling of other polymeric materials [20,33]. It should be remarked, however, the unusual trend that exhibits the diffusion coefficient, decreasing with the increase in temperature.

It is well known that diffusion coefficients have a dependence on temperature that is similar to the Arrhenius equation:

(3) $$D=D_{0}exp\left(-\frac{E_{D}}{RT}\right)$$

where *E*_D_ is the apparent activation energy for the diffusion process. For both aerogels, the expected linear dependence was obtained, but as a result of the inverse tendency that is shown by the diffusion coefficients with the temperature (Table 3), negative activation energy values were obtained. From a physical point of view, this fact indicates that there is another process competing with the diffusion, giving rise to negative values of this parameter. On the other hand, an analysis of the swelling values as a function of time, for each material at each temperature, according to the power law relation $W=kt^{n}$, allowed for finding the values of the release exponent, *n*, which are shown in the Table 3. Here, again, values are obtained that have no physical sense (for cylinders, *n* should be between 0.46 and 1) [34]. As seen above, both of the aerogels have a porous structure, with pore sizes ranging between 15 and 23 nm. According to these morphological characteristics, it should be expected that a Fickean-type diffusion kinetics would be fulfilled, since the relaxation of the polymer chains is not the limiting step for swelling. Consequently, the experimental data had to show a linear dependence between the swelling and *t*^½^. However, this is not the case either.

All of this analysis shows that along with the diffusion, another process besides swelling is taking place. In this sense, the Eyring equation [35,36] can provide information about the diffusion mechanism that takes place and sheds light on the causes of the observed negative values for the activation energies of the global diffusion process:

(4) $$k=\frac{k_{B}T}{h}\;exp\left(-\frac{\Delta G^{‡}}{RT}\right)$$

here *k*_B_, *h*, and *R* are the Boltzmann, Planck, and the gas constants, respectively. The activation Gibbs free energy, *ΔG*^‡^, is related to the activation enthalpy and entropy (*ΔH*^‡^, *ΔS*^‡^), according to the following expression:

(5) $$\Delta G^{‡}=\Delta H^{‡}-T\Delta S^{‡}$$

Then, substituting (5) in (4):

(6) $$k=\frac{k_{B}T}{h}\;exp\left(\frac{\Delta S^{‡}}{R}\right)\;exp\left(-\frac{\Delta H^{‡}}{RT}\right)$$

which can be linearized as follows:

(7) $$ln\frac{k}{T}=ln\frac{k_{B}}{h}+\frac{\Delta S^{‡}}{R}-\frac{\Delta H^{‡}}{R}\cdot \frac{1}{T}$$

This kinetic treatment describes the dependence of the rate of a chemical reaction with temperature when the concepts of statistical mechanics are applied. Even though this analysis is based on the theory of absolute reaction rates developed to treat ordinary chemical reactions, it was demonstrated that this model could be successfully used to describe the kinetic treatment of viscosity and diffusion [37,38].

In order to elucidate the mechanism that takes place during the diffusion of water through these aerogels using the statistical approach of Eyring, it is necessary to estimate the rate constants of the swelling process. The experimental data showed an excellent fit to the following Equation proposed by Schott [39]:

(8) $$\frac{t}{W}=A+Bt$$

which describes a second-order kinetics with respect to the unrealized swelling:

(9) $$\frac{dW}{dt}=k{\left(W_{\infty }-W\right)}^{2}$$

and the kinetic constant becomes equal to:

(10) $$k=\frac{1}{AW_{\infty }^{2}}$$

**Figure 8:**
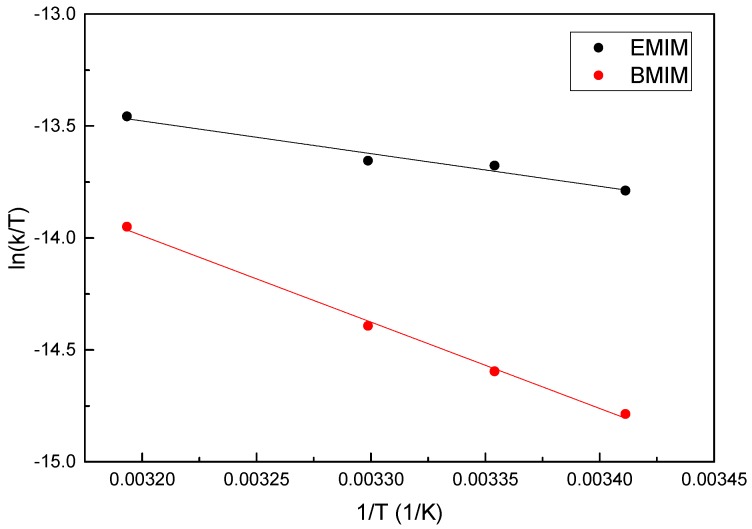
Plots of ln(k/T) versus 1/T using the second-order rate constants obtained from Equation (10) for the two aerogels.

The calculated values of the second-order rate constant, *k*, are included in Table 3, and were used to perform a thermo-kinetic analysis according to the Eyring Equation (7), as shown in Figure 8. The excellent adjustment that is obtained is evident. In Table 4, the values of *ΔH*^‡^, *ΔS*^‡^, and *ΔG*^‡^ are summarized.

When analyzing the thermo-kinetic parameters, it is observed that the activation enthalpies are positive due to the endothermic change from starting intermediates to the transition state during the overall diffusion process. According to these activation parameters, at 298.15 K, the change of the activation Gibbs free energy is largely controlled by the activation entropy term (e.g., −*TΔS*^‡^ = 81 kJ/mol for the CsE aerogel), while the contribution of the activation enthalpy to the transition state is not so significant (*ΔH*^‡^ = 12 kJ/mol). In this sense, it is important to note that these data indicate a significant increase in the order of the atoms that are involved during the pass to the transition state, with a small absorption of heat.

The values of these activation parameters are very similar to those that are observed during the formation of hydrogen bonds [40,41,42]. Their magnitudes coincide with the chemical nature of hydrogen bonding. Their formation involves relatively low energy and implicates the rearrangement of water molecules around the polymer, creating a hydration shell that is spatially oriented towards the hydroxyl and amino groups of chitosan.

At this point, it is important to remember that during the dissolution of chitosan in ionic liquids, the intra- and intermolecular hydrogen bonds of chitosan chains are broken. Although at the moment of forming the physical cross-linking the formation of some amount of hydrogen bonds is propitiated, these are relatively few in comparison with the large number of groups that are able to form hydrogen bonds in the polymer. In this way, during the swelling of the aerogels in water, along with the diffusion process, the generation of a huge amount of hydrogen bonds takes place with the consequent formation of a hydration shell. This analysis unambiguously supports the observed complex nature of the swelling processes of these aerogels, whose hydrated structure is apparently organized through hydrogen bonds. For this reason, a large contribution of *ΔS*^‡^ to the transition state is appreciated, when compared to that of *ΔH*^‡^.

## 4. Conclusions

Physical ionogels were obtained from chitosan solutions in EMIMAc and BMIMAc by non-solvent agent vapor diffusion (ethanol or water). The gels that were formed with ethanol treatment were rigid and brittle. Conversely, water vapor treatment only produce gels from the EMIMAc solution, these gels were too soft and brittle to be processed. Aerogels were produced by supercritical CO_2_ drying using ionogels that were produced by ethanol treatment. Such aerogels were low density mesoporous materials with surface area between 350 and 480 m^2^/g. There is neither spectroscopic evidence of changes in the chemical identity of the chitosan after aerogels production nor evidence of residual IL in the obtained materials. The internal structures of both types of aerogels were similar, appearing as mesoporous materials that are formed by agglomerated clumps.

The swelling of these aerogels followed a second-order kinetics. The application of the Eyring equation to the dependence of the rate constants on temperature allowed for clarifying the characteristics of the diffusion of water. The change of the activation Gibbs energy is mainly controlled by the activation entropy, rather than by the activation enthalpy at the tested temperatures. The kinetics of swelling seems to be controlled by the formation of hydrogen bonding, instead of the diffusion of water itself.

Aerogels with such characteristics have the potential for applications in the pharmaceutical industry as materials for the encapsulation, retention, and transport of model molecules that have affinities for chitosan. In the environmental field, these aerogels can be used as materials for the removal of pollutants from the effluents of industries in an extractive process by picking up minerals or compounds that are considered to be contaminants with affinity for chitosan. Another potential use is in the food industry or biotechnology, where components such as enzymes, proteins, or compounds that are part of a process could be immobilized inside the aerogels.

## Figures and Tables

**Table 1:** Outcome of the treatment of Cs-IL solutions with non-solvent agents vapor diffusion.

Cs-IL Solution	Non-Solvent Agent	
	Water	Ethanol
CsEMIM	Ionogel (soft)	Ionogel (CsE)
CsBMIM	dilution	Ionogel (CsB)

**Table 2:** Specific surface area (*S*_BET_), pore volume (*V*_p_) and pore diameter (*D*_p_) of the CsE and CsB aerogels.

Parameters	CsE	CsB
*S*_BET_ (m^2^/g)	358 ± 79	478 ± 264
*V*_p_ (cm^3^/g)	0.0733 ± 0.016	0.236 ± 0.083
*D*_p_ (nm)	31.0 ± 1.4	46.0 ± 3.6

**Table 3:** Values of the degree of swelling, diffusion coefficient, second-order kinetic constant and the release exponent of CsE and CsB aerogels of swelled in water at different temperatures.

*T* (°C)	CsE				CsB			
	*W*_∞_ (g H_2_O/g gel)	*D* × 10^10^ (m^2^/s) ^a^	*k* × 10^4^ (s^−1^) ^b^	*n* ^c^	*W*_∞_ (g H_2_O/g gel)	*D* × 10^10^ (m^2^/s) ^a^	*k* × 10^4^ (s^−1^) ^b^	*n* ^c^
20	4.64	3.99	3.011	0.16	6.56	3.36	1.111	0.26
25	3.96	3.55	3.426	0.20	5.22	3.32	1.367	0.31
30	3.79	3.01	3.464	0.23	6.43	3.09	1.703	0.35
40	3.59	2.54	4.481	0.29	5.15	2.68	2.738	0.37

^a^ Evaluated according to Equation (2), ^b^ Evaluated according to Equations (8) and (10), ^c^ Dimensionless release exponent from the power law relation.

**Table 4:** Activation energies calculated from *D* by the Arrhenius Equation (3). Thermo-kinetic parameters: *ΔH*^‡^ and *ΔS*^‡^ estimated from the Eyring Equation (7) and *ΔG*^‡^ calculated at 298.15 K using the Equation (5).

Aerogel	*E*a (kJ/mol)	*ΔH*^‡^ (kJ/mol)	*ΔS*^‡^ (J/mol·K)	*−TΔS*^‡ a^ (kJ/mol)	*ΔG*^‡ a^ (kJ/mol)
CsE	−17.5	12.1	−271	80.7	92.9
CsB	−9.2	32.1	−211	63.0	95.1

^a^ Evaluated at *T* = 298.15 K.
